# TranSegNet: Hybrid CNN-Vision Transformers Encoder for Retina Segmentation of Optical Coherence Tomography

**DOI:** 10.3390/life13040976

**Published:** 2023-04-10

**Authors:** Yiheng Zhang, Zhongliang Li, Nan Nan, Xiangzhao Wang

**Affiliations:** 1 Laboratory of Information Optics and Opto-Electronic Technology, Shanghai Institute of Optics and Fine Mechanics, Chinese Academy of Sciences, Shanghai 201800, China; zhangyiheng@siom.ac.cn (Y.Z.);; 2 Center of Materials Science and Optoelectronics Engineering, University of Chinese Academy of Sciences, Beijing 100049, China

**Keywords:** optical coherence tomography, image segmentation, vision transformer, convolutional neural network

## Abstract

Optical coherence tomography (OCT) provides unique advantages in ophthalmic examinations owing to its noncontact, high-resolution, and noninvasive features, which have evolved into one of the most crucial modalities for identifying and evaluating retinal abnormalities. Segmentation of laminar structures and lesion tissues in retinal OCT images can provide quantitative information on retinal morphology and reliable guidance for clinical diagnosis and treatment. Convolutional neural networks (CNNs) have achieved success in various medical image segmentation tasks. However, the receptive field of convolution has inherent locality constraints, resulting in limitations of mainstream frameworks based on CNNs, which is still evident in recognizing the morphological changes of retina OCT. In this study, we proposed an end-to-end network, TranSegNet, which incorporates a hybrid encoder that combines the advantages of a lightweight vision transformer (ViT) and the U-shaped network. The CNN features under multiscale resolution are extracted based on the improved U-net backbone, and a ViT with the multi-head convolutional attention is introduced to capture the feature information in a global view, realizing accurate localization and segmentation of retinal layers and lesion tissues. The experimental results illustrate that hybrid CNN-ViT is a strong encoder for retinal OCT image segmentation tasks and the lightweight design reduces its parameter size and computational complexity while maintaining its outstanding performance. By applying TranSegNet to healthy and diseased retinal OCT datasets separately, TranSegNet demonstrated superior efficiency, accuracy, and robustness in the segmentation results of retinal layers and accumulated fluid than the four advanced segmentation methods, such as FCN, SegNet, Unet and TransUnet.

## 1. Introduction

Optical coherence tomography (OCT) is an influential biomedical optical imaging technology based on the principle of low coherence interference of light. The analysis of the interference information of backscattered light can facilitate noncontact and high-resolution imaging of the internal microstructure of the tissue [1]. In ophthalmology, various retinal diseases and conditions [2,3], such as age-related macular degeneration (AMD), diabetic retinopathy, and glaucoma, may cause morphological changes in the retina, including layer thinning or thickening, retinal tear, localized area bulging, and fluid leaks. Longstanding morphological changes can lead to functional damage, metamorphopsia, and decreased visual acuity. OCT can provide a cross-sectional high-resolution view of the retina and measure retinal blood velocity and flow with arbitrary orientation in vivo, making it a powerful diagnostic technique in ophthalmology [4]. Thus, reliable segmentation is crucial for OCT retinal image processing. Segmentation of the retinal layers and diseased tissues enables the accurate acquisition of the distribution and thickness of retinal structures, essential for clinical decision making and the prevention and diagnosis of related ophthalmic diseases.

OCT retinal segmentation is a challenging task. The unavoidable speckle noise in the OCT imaging system makes the originally continuous and clear tissue structure appear grainier and difficult to distinguish. The existence of blood vessels and motion artifacts caused by eye movements can lead to discontinuities and loss of detail between adjacent retina layers [5]. Additionally, as the imaging depth increases, the absorption or scattering of light by the retinal layer affects the signal-to-noise ratio (SNR) of the image, and the boundary between adjacent layers of the retina is highly diffused. Furthermore, manual hierarchical labeling by experts, based on experience, is subjective and time consuming. Therefore, automatic segmentation algorithms for OCT retinal images have constantly been the focus of ophthalmic OCT research.

For retinal OCT B-scan images, segmentation algorithms are typically divided into two categories [6]: retinal layer boundary and retinal layer extraction. Most early algorithms focused on fitting the retinal layer boundary based on image intensity and its derivatives. Ishikawa [7] proposed a novel preprocessing method for OCT images and used an adaptive threshold to determine the actual location of the boundary based on the reflectivity histogram of each A-scan. In 2008, Tan et al. [8] utilized dynamic programming with 2D gradient information to extract information and revealed the relationship between the thickness of the inner layer of the retina and the incidence of glaucoma. In 2012, Zhang et al. [9] proposed a segmentation algorithm that requires A-lines alignment, limiting its application to severe eye diseases such as AMD and glaucoma. Segmentation based on thresholding is significantly affected by image speckle noise and intensity discontinuity. Therefore, numerous researchers are now committed to improving the stability of algorithms, such as edge-detection [10]. Fernandez et al. [11] used the active contour method (Snake) to extract the fluid regions in the retina structure of patients with AMD. In 2009, Azadeh et al. [12] used a circular shape prior and a multi-phase framework to adapt Chan-Vese’s active contours for intra-retinal layer segmentation in the presence of low contrast and high noise. In 2011, Ghorble [13] designed a global-based segmentation method that uses a Kalman filter to simulate and detect the approximate parallelism of the retinal layer, combines local information with global information and then extracts eight layers of retinal boundaries based on active contours. The active contour model can find local optima, but its accuracy relies on the initial point being close enough. The graph-theoretic segmentation technique has recently been introduced into ophthalmic SDOCT segmentation applications, and has proven to be a successful technique [14]. Based on a geometric graph and surface constraints, Mona Haeker [15] developed an automated method for the automated segmentation of the internal limiting membrane and the pigment epithelium in 3-D OCT retinal images. In 2010, Chiu et al. [16] presented an automatic approach for segmenting layered structures in ocular images using graph theory and dynamic programming, with a focus on retinal layer segmentation in SDOCT images. The proposed technique significantly reduces processing time and allows for retinal layer thickness calculations, which are important for the detection of ocular diseases. In 2017, Mohandass [17] proposed a novel segmentation algorithm called Boisterous Obscure Ratio (BOR) with the Robust Outlyingness Ratio (ROR) denoising technique as its basis. BOR was developed to address the challenges in retinal diagnosis caused by the noise. Ma et al. [18] proposed a combination of structural interpolation and lateral averaging (SI-LMF) to improve the SNR based on single retinal image segmentation. They performed structural interpolation to eliminate retinal thickness fluctuations and successfully extracted ten layers of retinal boundaries from small datasets. In 2021, Shirokanev [19] proposed a 3D fundus structure model based on processing of OCT images and using approximating functions to describe the boundaries of the retina, which can help in selecting effective methods of treatment for diabetic retinopathy. In 2022, Liu [20] proposed an approach based on the improved Canny operator for automatic segmentation of retinal boundaries, capable of distinguishing eleven retinal boundaries without human intervention. However, its accuracy decreases for severe AMD patients with subretinal fluid and retinal structural deformation. Overall, traditional retinal segmentation algorithms have been widely used in ophthalmology for the identification and delineation of retinal layers, which can obtain smooth retinal layer boundaries. However, image noise, irregularities of eye motion and lesion structures challenge the mechanism of the algorithm and its generalization ability. Additionally, traditional algorithms often require manual adjustment and tuning, mostly requiring repeated calculations, leading to high computational requirements.

Automatic medical image analysis has made substantial progress in the past few decades as machine learning techniques have rapidly evolved. This progress has permeated the field of automatic retinal OCT segmentation. The retinal segmentation problem is gradually transformed into retinal layer extraction, where pixels of different retinal layers are assigned to different classes by semantic segmentation. In 2007, Zawadzki proposed a modified support vector machine (SVM) for semi-automatic segmentation of retinal layers and structures, and successfully tested the software in clinical settings for assessing both healthy and diseased retinal structures [21]. Similarly to Zawadzki et al., in 2011, Vermeer et al. [22] used a support vector machine (SVM) to learn and classify each pixel and used regularization to smooth the classification interface, which improved the robustness of the algorithm. However, owing to the limitations of SVM, multiple models must be combined. U-Net [23], proposed in 2015, has been validated for retinal segmentation and outperforms SVM. In 2016, SegNet [24] was proposed by Vijay et al. It is distinguished by its decoder, which upscales lower-resolution input feature maps with pooling indices from the corresponding encoder, resulting in fewer parameters, memory needs, and training time. In 2017, ROY et al. [25], inspired by U-Net and DeconvNet [26], proposed the fully convolutional framework RelayNet for end-to-end retinal layers and fluid segmentation. DeepRetina [27] proposed in 2020 used a modified Xception65 to extract and learn the features of the retinal layer and input it to atrous spatial pyramid pooling (ASPP) to obtain multi-scale feature information and complete the automatic segmentation of retinal layers using a decoder. In 2021, Yadav et al. [28] proposed a cascaded two-stage network based on two compressed U-nets, where the first network was responsible for segmenting retinal tissue from OCT B-scans, and the second network segmented eight inner retinal layers with high fidelity. In 2022, Fazekas et al. [29] proposed a spatially decomposed layer segmentation network (SD-LayerNet), which is a fully convolutional semi-supervised retinal layer segmentation method customized with a set of prior retinal information encoded as self-supervised loss terms. Despite the remarkable characterization capabilities of CNN-based methods, their ability to recognize pathological structures with significant differences in texture, shape, and size is limited in their ability to extract visual patterns across different spatial positions.

Retina OCT segmentation methods based on pixel-based classification algorithms are more intuitive to visualize, allowing individual segmentation of lesion tissues that can be measured using clear metrics. However, the uneven distribution among the retinal layers and lesion tissue areas contributes to the reduced attention of these methods to poor frequency categories (such as accumulated fluid), resulting in misclassification and logical confusion [6]. The transformer [30], with global self-focus mechanisms, is considered a viable alternative to CNNs, and the vision transformer (ViT) [31] is a transformer targeted at vision processing tasks such as image recognition. Unlike CNNs, which expand the receptive field using convolutional layers, ViT has a larger view window, even at the lowest layer. TransUNet [32], proposed by Chen in 2021, is the first transformer-based medical image segmentation framework that builds on the highly successful ViT, which establishes self-attention mechanisms from the perspective of sequence-to-sequence prediction. The method integrates both transformers and CNNs in the encoder, utilizing the strengths of each architecture to improve performance. Although such CNN-Transformer models have shown great promise for computer vision-processing tasks, there are still some challenges, including dependence on a large amount of labeled data to achieve optimal performance and high computational requirements due to a large number of parameters and complex architecture. These factors can impact its performance and usability in certain applications where labeled data are difficult or expensive to obtain with resource-limited deployment.

To address these problems, we proposed a hybrid CNN-ViT network, named TranSegNet for retinal OCT image segmentation. Combining the upgraded CNN backbone network and a lightweight design of ViT with convolutional attention, TranSegNet can be applied effectively to small-scale datasets without pre-training. The network backbone in TranSegNet is based on an upgraded U-shaped network to enhance spatial information, which detects multi-scale resolution feature information using CNNs. Incorporated ViT at the end of the CNN-encoder part, TranSegNet introduces the multi-head attention mechanism to improve global modeling ability by encoding image features as sequences. We produced a healthy retinal dataset from the SD-OCT system built in our lab for model training and additionally trained our model using a publicly available retinal dataset of DME patients for more comprehensive validation of the model performance. The evaluation metrics demonstrated that our model achieved accurate segmentation of retinal structures for healthy and pathological retinal OCT images, outperforming four state-of-the-art methods.

## 2. Methods

### 2.1. Problem Statement

Each frame of the OCT retinal B-scans x is defined as follows: x ∈ R^C×H ×W^, the image resolution is H × W, and C is the number of channels. The goal is to predict the corresponding pixel-label mapping of size H × W by assigning each pixel to a specific label L. We consider the current segmentation task to be a K-classification problem.

**Figure 1:**
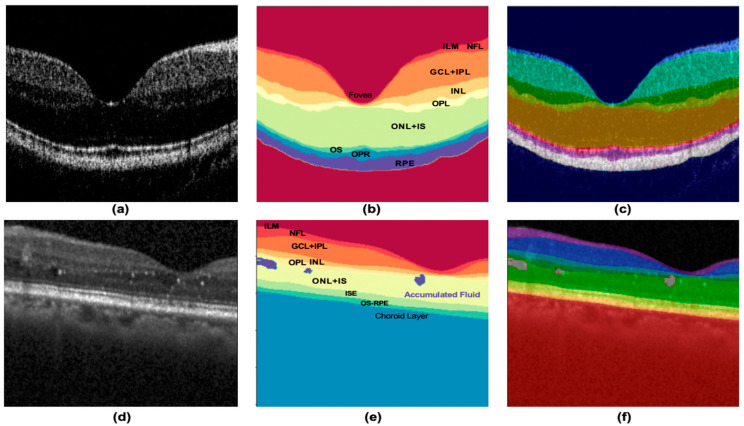
Demonstration of (**a**–**c**) original retinal B-scan images of Dataset A, labels, and their definitions, respectively; (**d**–**f**) original retinal B-scan images of Dataset B, labels, and their definitions, respectively. Abbreviations: ILM Inner limiting membrane, NFL—Nerve fiber layer, GCL—Ganglion cell layer, IPL—Inner plexiform layer, INL—Inner nuclear layer, OPL—Outer plexiform layer, ONL—Outer nuclear layer, IS(ISE)—Inner segments of the photoreceptors, OS—Outer Segments of the photoreceptors, OPR—Outer photoreceptor, RPE—Retinal pigment epithelium.

This study used two retinal OCT datasets to evaluate the model comprehensively. Dataset A was obtained from healthy retinal images provided by the SD-OCT system in our laboratory. Figure 1a,b shows the original OCT image of Dataset A and its label definition. The segmentation target is to extract the eight layers of the retina marked in Figure 1c; therefore, the final output of the model is K = 9 (plus background). Dataset B was obtained from the Duke SD-OCT publicly available dataset of DME patients [33,34]. Figure 1d,e shows the original OCT image of the retina of Dataset B and its label definition. The segmentation target was to extract the eight layers of the retina and the accumulated fluid marked in Figure 1c with a final output K = 10.

### 2.2. Network Architecture

**Figure 2:**
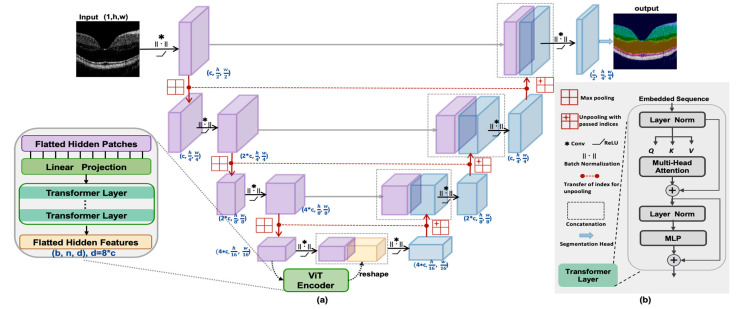
(**a**) Structure of the proposed TranSegNet (**b**) Structure of the Transformer layer.

The network structure of TranSegNet is shown in Figure 2a. It consists of a contracting path composed of encoder blocks, an expansive path composed of decoders, and a segmentation head. The backbone uses cascade layers to pass features of different resolutions from the encoder blocks to the matching decoder blocks and finally to the classification output. The details of each component are as follows:

#### 2.2.1. CNN-Transformer Hybrid as an Encoder

CNN feature extraction. In the encoder section, TranSegNet takes the form of a CNN-ViT hybrid architecture in which the CNN is first used as a feature extractor to generate an input feature-mapping sequence. Each encoder contains the following layers: a 3 × 3 convolutional layer, a normalization layer, a ReLU layer, and a maximum pooling layer. The first encoder performs convolutions with step = 1 twice and then once with a step = 2 convolution layer. In the other encoders, convolutions with step = 1 were executed twice. These small convolutions can help the network introduce additional ReLU layers to improve nonlinear representability [35]. At the end of the CNN-encoder part, ViT with multi-head attention is incorporated to extract important feature maps.

Image serialization. Because the input of the ViT encoder is a sequence different from the size of the image feature map, image serialization is a necessary step by dividing the extracted CNN feature map of size C × H × W into n square patches (*p, p, c*) in raster order (left to right, top to bottom), where *p* is a predefined parameter indicating the size of the patches. The flattened patches are multiplied with a trainable embedding tensor E of shape (*p^2^ · c, d*) to be linearly projected to dimension d, finally obtaining the sequence X:

(1) $$X=\left[x_{class};x_{p}^{1}E;x_{p}^{2}E;\ldots ;x_{p}^{n}E\right],E\in R^{\left(p^{2}\cdot c\right)\times d}$$

Compared to traditional Transformer models, our VIT design differs in that we have removed the extra position encoding for the lightweight design, which aims to balance model performance with computational efficiency and practical considerations. The ViT encoder extracts patches from the CNN feature map rather than directly from the original image, which allows the model to fully utilize the CNN feature map. The literature [19,22] states that a hybrid CNN-transformer encoder performs better than using a transformer independently as an encoder.

**Figure 3:**
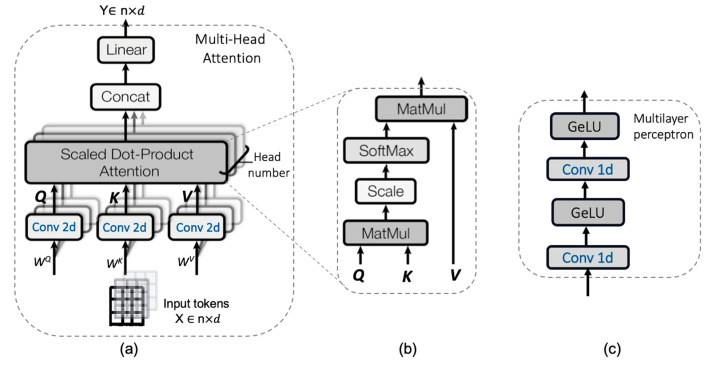
Components of the transformer (**a**) multi-head attention, (**b**) scaled dot-product attention, and (**c**) multi-layer perceptron.

Transformer. The transformer layer [23,24] contains the multi-head attention (MHA) mechanism and a multilayer perceptron (MLP) layer, as well as layer normalization and residual connectivity, as shown in Figure 2b. The core of the transformer is a multi-head self-attention mechanism, as shown in Figure 3a. The input *X* is a sequence of dimensions *X* ∈ R^n×d^, where n is the sequence length and each sequence has a feature dimension of d. The input is then equally divided according to the head number in the feature dimension to obtain *X_i_* ∈ R ^(n)×di^, where i = 1, 2, 3, ..., h, and h represents the head number. In the lightweight design of ViT, we use 2D convolution operations to replace linear projection to generate three matrices *Qi* (query)*, Ki* (key), and *Vi* (value) with three trainable matrices *W^Qi^*, *W^Ki^*, and *W^Vi^* of the shape (dₕ, dₕ), thus improving the model in performance and efficiency by introducing convolutions into ViT [35]:

(2) $$Q_{i}={Conv\;2D(W}^{q}\left(Xi\right))$$

(3) $$K_{i}={Conv\;2D(W}^{k}\left(Xi\right))$$

(4) $$V_{i}={Conv\;2D(W}^{v}\left(Xi\right))$$

where *Q*_i_, *K*_i_, and *V*_i_ represent the projections of the input in the three subspaces. Subsequently, the weights of the features are learned using scaled dot-product attention, as shown in Figure 3b, which first calculates the similarity between *Q*_i_ and *K*_i_ to obtain *A*_i_. Subsequently, the final “attention” *SA*_i_ is calculated as follows:

(5) $$A_{i}=\mathrm{softmax}\left(\frac{Q_{i}\cdot k_{\dot{l}}^{T}}{\sqrt{d_{h_{d}}}}\right)$$

(6) $$SA_{i}=A_{i}\cdot V_{i}$$

Thus, we obtain *h* number of outputs *SA*_i_, concatenate them along the feature dimension, and process them through a linear layer to obtain the final layer output *Y*. We designed the multilayer perceptron shown in Figure 3c as the classification output and then completed one round of transformer coding after two fully connected convolutional layers.

#### 2.2.2. Decoder

The decoding block involves five main layers: unpooling, cascade, convolution, batch normalization, and the RELU activation function. The unpooling layer upsamples the previous low-resolution level to a finer resolution using the saved position indices from the corresponding encoder block. Such an unpooling layer ensures that the spatial information remains preserved, in contrast to interpolation-based upsampling. This is particularly important for accurately segmenting the layers near the foveal region of the retina, as they typically have only a few pixels, and bilinear interpolation may lead to highly diffuse boundaries, leading to unreliable layer thickness estimation. In the cascade layer, the upsampled features were concatenated with the output features of the matched encoder in the contraction path. The kernel size of the convolutional layer was 3 × 3, which is consistent with that of the encoder, and convolutions with a step size of 1 were performed twice in each decoder. The second part is the segmentation head, which maps feature maps to K-channel feature maps (for K classes) through a convolutional layer with a 1 × 1 kernel.

The decoder blocks together with the hybrid encoder form a U-shaped architecture that enables feature aggregation at different resolution levels via skip connections. The detailed architecture of TranSegNet and the intermediate skip connections are shown in Figure 2.

### 2.3. Loss Function

The cross-entropy loss function is often used in neural network classification problems to evaluate the proximity of the actual output to the predicted output in terms of a probability distribution. However, it computes the average losses on a per-pixel basis, which is considered discrete. The greater the imbalance in the label distribution, the more challenging training becomes. Therefore, this study weighted the cross-entropy loss function based on the areas of different categories in the retinal image. The function is defined as follows:

(7) $$L_{ce}=-\sum_{l=1}^{k}w_{l}\left(x\right)g_{l}\left(x\right)\log p_{l}\left(x\right)$$

where $p_{l}\left(x\right)$ represents the estimated probability that pixel *x* belongs to category *L* and $w_{l}\left(x\right)$ is the weight associated with pixel *x*. $g_{l}\left(x\right)$ represents the actual probability that pixel *x* belongs to category *L*. Despite the weighted cross-entropy loss function mitigating imbalanced categories, it remains insufficient for global training enhancement and cannot address the inbuilt issue of cross-entropy loss. Therefore, the Dice loss function $L_{dice}$ is added to this study to measure the similarity between the predicted output and ground truth. It is particularly useful for image segmentation tasks as it measures the overlap between the predicted output and the ground truth [36].

(8) $$L_{dice}=1-\frac{2\Sigma_{x\in \Omega }p_{l}\left(x\right)g_{l}\left(x\right)}{\sum_{x\in \Omega }p_{l}^{2}\left(x\right)+\sum_{x\in \Omega }g_{l}^{2}\left(x\right)}$$

Finally, TranSegNet was trained by combining the upper loss functions by introducing the weight parameters α and β. The final $L_{overall}$ is shown as:

(9) $$L_{overall}={\alpha L}_{ce}+{\beta L}_{dice}$$

## 3. Experimental Setup

### 3.1. Dataset

**Figure 4:**
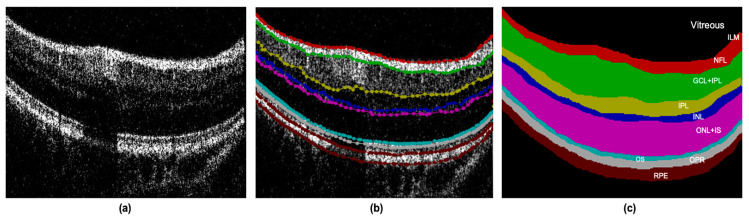
Description of retinal OCT dataset A. The original retinal OCT B-scans (**a**) are manually sampled with approximately 80 to 100 points for each boundary, forming closed curves after which the retinal layers were extracted, shown as (**b**); (**c**) the manually labeled image and its label annotation; different colors represent different layers.

In this study, two retinal OCT datasets were used to evaluate the performance of the model in various applications. Dataset A was sourced from a laboratory-built SD-OCT system. The light source used in our system was a super-radiant light-emitting diode (SLED) with a central wavelength of 840 nm, half-height bandwidth of 50 nm, and in-air axial resolution of 7.4 μm. Each set of acquired retinal OCT images contained 280 volumes, for a total of 11,200 images (40 B-scans per volume), with a size of 280 × 400. OCT scans of the healthy retinas of several volunteers were performed with 20 frames on each side of the fovea (±1, ±2, ..., ±20 on each side of the central fovea). Figure 4a shows an OCT image of the retina. We filtered the data and selected a total of 14 groups equally and proportionally from the 280 groups, each containing the eye foveal scan 𝑘_𝑓𝑜𝑣_ and the scan 𝑘_𝑓𝑜𝑣_ ± 1, 𝑘_𝑓𝑜𝑣_ ± 10, 𝑘_𝑓𝑜𝑣_ ± 15 and 𝑘_𝑓𝑜𝑣_ ± 20, and finally obtained 200 OCT retina B-scans. The selected B-scans were labeled under the guidance of professional clinicians, as shown in Figure 4b, where each retinal layer has its unique area color and label designation as shown in Figure 4c. Each image is labeled with eight layers of retinal structures, which completes the retinal OCT image database Dataset A.

Given the lack of pathological retinal samples in Dataset A, the model was trained with the Duke public SD-OCT dataset [34] as Dataset B. Dataset B contains 110 annotated SD-OCT B-scan images from 10 DME patients (11 B-scans per patient), each with a frame size of 512 × 740. The selected images for each patient include the eye foveal scan 𝑘_𝑓𝑜𝑣_ and the scan 𝑘_𝑓𝑜𝑣_ ± 2, 𝑘_𝑓𝑜𝑣_ ± 5, 𝑘_𝑓𝑜𝑣_ ± 10, 𝑘_𝑓𝑜𝑣_ ± 15, and 𝑘_𝑓𝑜𝑣_ ± 20, and two ophthalmologists manually segmented all fluid-filled regions and eight retinal layer boundaries.

We used data argument owing to the relatively small sample size of the dataset. First, a data-slicing operation with a random window was performed for each original OCT B-scan based on a sliding window of size 256 × 256. Data slicing is guided by the following two principles: (1) the position of each slice is different to ensure that each slice contains different information; (2) when conducting the slicing, all slices generated from the original image can cover all the feature areas of the original image. During the training process, random image contrast and brightness adjustments were added, and random erasing was applied to simulate the effects of blood vessels in the retinal layer.

### 3.2. Experimental Settings

Given the limited scale of labeled retinal image datasets, we employed cross-validation by dividing the dataset into k folds (k = 5) in this study to train and evaluate the model. We divided the self-made Dataset A and Dataset B into a training set and validation set in a ratio of 4:1. In each iteration of the cross-validation process, four folds were used for training and one fold for validation. After repeating this process five times, we averaged the results across the iterations to obtain a more reliable estimate of the segmentation performance. To balance the contribution of each term in the overall training, the parameters of the loss equation are set to α=β=0.5 in Equation (9). The weights are calculated based on the frequency of each class in the training set, with higher weights assigned to classes representing low-frequency regions, such as lesion areas in retinal OCT images. The weights are then directly applied to the loss function during training, allowing the model to place greater emphasis on correctly classifying low-frequency regions. The Adam optimization algorithm [37] is adopted in this study to replace the traditional stochastic gradient descent method in the training process. Specifically, the algorithm calculates the exponential moving averages of the gradients and squared gradients, and parameters β1 and β2 control the decay rate of these moving averages. The Adam configuration parameters were set as follows: learning rate = 0.001, β1 = 0.9, β2 = 0.999, and epsilon = 1 × 10^−8^. All the samples in the dataset were randomly formed into a batch size of 8. By monitoring the curves of the loss function in the validation set, we set the total number of training epochs to 50 and referred to the accuracy of each validation set to select the final model to be deployed. The overall process was performed on a laboratory workstation equipped with an NVIDIA RTX3070 GPU and Intel Core i7-10700 CPU based on the PyTorch deep learning framework.

### 3.3. Evaluation Metrics

The choice of performance evaluation metrics in this study was motivated by the task of OCT retinal segmentation, which involved predicting the category of each pixel in the output image. The predicted image’s shape matched the spatial resolution of the input image, and its channel depth was equal to the number of categories to be predicted. To evaluate the performance of the proposed methods, we used several widely recognized metrics, including accuracy (Acc), precision, recall, dice similarity coefficient (DSC), and Hausdorff distance (HD), as suggested by previous studies [38,39]. Accuracy, defined as Equation (10), directly reflects the proportion of correct results predicted by the model, and we determined the optimal model by calculating accuracy during the training process. Precision and recall in Equations (11) and (12) are complementary metrics that provide information on the quality of the predictions, particularly in the case of imbalanced datasets. Precision effectively reflects the purity of our positive detections relative to the ground truth, while recall describes the completeness of positive predictions relative to the ground truth. DSC is a widely used metric for evaluating the overlap between the ground truth and predicted output, which quantifies the percentage of overlap between them and provides their similarity. DSC is particularly useful for evaluating the segmentation accuracy of medical images, as it measures both true positive and false positive predictions. It is defined as Equation (13):

(10) $$Acc=\frac{TP+TN}{TP+FN+FP+TN}$$

(11) $$Precision=\frac{TP}{TP+FP}$$

(12) $$Recall=\frac{TP}{TP+FN}$$

(13) $$DSC=\frac{2\;*\;TP}{2\;*\;TP+FP+FN}$$

where true positive (TP) indicates correctly segmented retinal layer pixels, false positive (FP) indicates incorrectly predicted nonretinal layer pixels, false negative (FN) indicates incorrectly segmented retinal layer pixels, and true negative (TN) indicates correctly segmented nonretinal layer pixels. These four parameters are more sensitive to the distribution of pixels within the retinal layer; therefore, this study also adopted the HD as a definition of the distance between two-point sets as a metric for assessing the segmented retinal layer boundary, defined as follows:

(14) $$H\left(A,B\right)=\max\left(h\left(A,B\right),h\left(B,A\right)\right)$$

Equation (14) represents a two-way Hausdorff distance, which is the most basic form of the HD, where

(15) $$h\left(A,B\right)=\underset{a\in A}{max}\left\{\underset{b\in B}{min}\left\|a-b\right\|\right\}$$

(16) $$h\left(B,A\right)=\underset{b\in B}{max}\left\{\underset{a\in A}{min}\left\|b-a\right\|\right\}$$

*h*(*A, B*) and *h*(*B, A*) are the one-way HDs from set *A* to set *B* and from set *B* to set *A*, respectively. From Equation (14), the two-way *H*(*A, B*) is larger than the two one-way distances *h*(*A, B*) and *h*(*B, A*), and measures the maximum mismatch between the two point sets. This metric is useful in assessing the overall boundary accuracy of the predicted segmentation, which is critical for accurate diagnosis and treatment planning in medical imaging.

## 4. Experimental

### 4.1. Comparison of TranSegNet with Comparative Methods

We performed experiments to compare the proposed model with other state-of-the-art segmentation methods, including FCN [40], Unet [23], SegNet [24] and TransUnet [32]. To ensure that the comparison experiments were as fair as possible, we adapted the layers of the encoder and decoder, the size of the convolutional kernel, and the number of channels according to the above comparison methods. For TransUnet, which also contains the ViT module, we kept its parameters consistent with those of our proposed model and reduced its layer depth.

**Figure 5:**
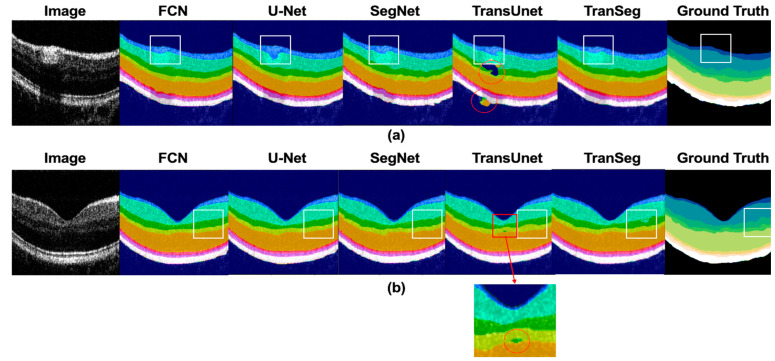
Comparison results of (**a**) retinal segmentation under the influence of blood vessels and (**b**) segmentation of retina near the fovea in Dataset A.

**Figure 6:**
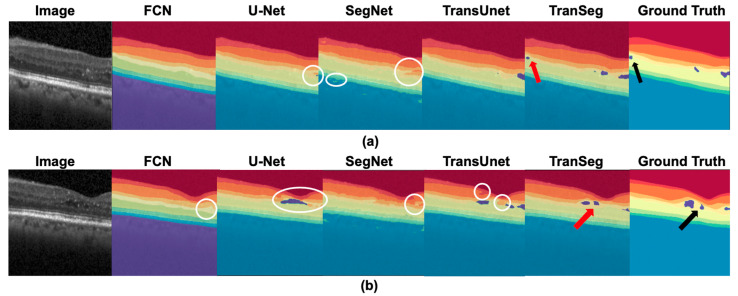
Comparison results of the pathological retina of DME patients (**a**) segmentation with macular edema (ME) (**b**) segmentation with accumulated fluid near the fovea in Dataset B. The red arrow indicates the segmented ME area, while the black arrow indicates the actual ME area.

Considering the characteristics of the two datasets, two qualitative comparison methods were designed. Comparison of retinal B-scan image segmentation results with significant vascular influence (Figure 5a) and retinal images near the fovea (Figure 5b) is shown in Dataset A. In Dataset B, we used pathological retinal images of a patient with DME (Figure 6a) and images with fluid in the fovea of the macula to compare segmentation results (Figure 6b). 

The presence of blood vessels in the retina significantly impacted the detection of deep interferometric signals, leading to the discontinuity of the layer in retinal OCT B-scans. Therefore, we examined their ability to infer and segment accurately in this case. The segmentation results in Figure 5a demonstrate that TranSegNet outperformed other methods in terms of segmentation accuracy, especially in areas where the influence of blood vessels is significant. From the white boxes highlighted in Figure 5a, it is evident that TranSegNet can accurately segment the NFL and GCL+IPL, which are vulnerable to the influence of blood vessels, whereas other methods exhibited varying degrees of pixel misallocation. In addition, TransUNet showed more pixel errors in segmentation without pre-training, which are highlighted in red circles in Figure 5a. The relatively thin layer in the central fovea region of the retina also presents a challenging segmentation situation. As shown in Figure 5b, TranSegNet successfully restored more details in the fovea area of the retina B-scan, while other methods segmented retinal layers with loss of edge details, as shown in the white box. Therefore, our method demonstrates better performance in Dataset A. 

Figure 6 shows the segmentation results of two types of DME diseased retinas in Dataset B. As shown in the figure, FCN and SegNet are insensitive to the accumulation fluid areas and are unable to identify them, while U-net and SegNet extracted retinal layers with isolated mislabeling and the phenomenon of edge burr. The white circles in Figure 6 highlight the inference errors. TransUnet and TranSegNet extracted both the retinal layers and accumulation fluid; however, TranSegNet was more accurate in locating and sizing the fluid area indicated by the red arrow in Figure 6. In conclusion, our method demonstrates superior accuracy in segmenting the retina with pathological changes.

Dataset A, Comparing results. Table 1 lists the experimental metrics for the selected optimal models after training using different methods. We calculated the average results over the five cross-validation runs to evaluate the quantitative results. TranSegNet reached an accuracy of 94.64% in the first 50 rounds, higher than the other methods, with a value of the loss function equal to 0.1578. The loss function of our model was reduced to 0.20 by the 6th round of training, whereas the loss functions of the other methods barely converged to lower than 0.2 in the first 50 epochs of training, indicating that TranSegNet learns the information of features quickly and efficiently. Comparing the average HD for all retinal layers after segmentation, the boundaries of the retinal layers predicted by TranSegNet were closer to the actual boundaries, suggesting a higher sensitivity to the retinal layer boundaries. In the training process of Dataset A, we also calculated the FLOPs, which stand for “Floating Point Operations,” to measure computational complexity. The results showed that our model has a complexity second only to FCN. The number of parameters for all methods and the total duration of 50 training epochs are recorded in Table 1, which showed the parameters of TranSegNet are reduced by more than half compared to TransUNet and the average training time required is also less than other methods. This demonstrates that through the lightweight design, our model achieves a great reduction in computational cost without sacrificing performance, which is efficient and effective for practical applications.

**Figure 7:**
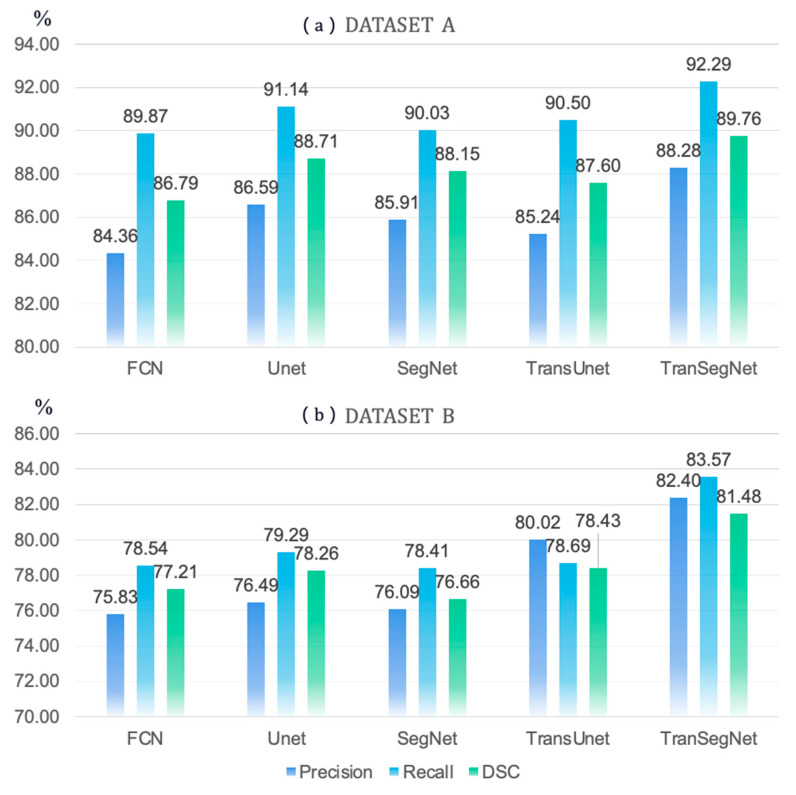
Comparison of Average Precision, Recall and DSC of Segmentation results by Different Methods on Dataset A (**a**) and Dataset B (**b**).

Table 2 lists the metrics for each class in the segmentation output of OCT retinal images in the test set of Dataset A. Compared with the other methods, TranSegNet reached optimal values in the three metrics of precision, recall, and DSC, most often (bold in the table) for recognizing each layer of the retina, and the metrics were evenly distributed. The metrics for evaluating GCL + IPL and INL were the best among all the methods for retinal scans near the fovea region. As shown in Figure 7a, the bar chart compares the average precision, accuracy, and recall of all the categories in segmenting retinal OCT images using different methods, where different methods perform relatively similarly in segmenting dataset A. However, our model achieved the best performance and consistency in all three metrics than other models, which shows that TranSegNet achieved outstanding and balanced segmentation performance for all categories on Dataset A.

Dataset B, Comparing results. Dataset B has feature information on the retinal image, with eight layers of the retina and fluid accumulation area as segmentation targets. The loss function of TranSegNet converged to 0.2087 in the validation set in the 19th training round, at which point the model achieved an accuracy of 95.20%. We also calculated the average to evaluate the quantitative results. Table 3 shows that our model outperforms other methods in more complex tasks. Among 30 indicators, including Precision, Re, and DSC, for 10 classifications, TranSegNet achieved 24 top scores (highlighted in bold in the table). Our model performed exceptionally well in identifying pathological conditions such as retinal layers with lesions and fluid accumulation, proved by the metrics of GCL+IPL, INL, OPL, ONL+IS, and fluid. Figure 7b demonstrates that our proposed model achieves superior performance in terms of average precision, recall, and DSC scores for segmenting pathological retinal structures. This is attributed to the model’s advanced convolutional attention mechanism for incorporating image features and spatial information. In summary, our proposed model has demonstrated advancements in retinal image segmentation, with improved performance in complex scenarios and superior accuracy in identifying and localizing retinal abnormalities.

### 4.2. Discussion

The TranSegNet framework proposed in this study is built on the core of the CNN-ViT hybrid encoder. ViT splits the feature maps from the CNN into small image patches and then feeds linear embedding sequences of these patches as input to the transformer while using the learnable embedding vector class token for the prediction of image classification to extract more powerful feature maps. In this study, we performed further research-based analysis of ViTs.

We referred to ViT [31] to set the size of the image blocks in the input sequence to 16 × 16. This study examined the effect on the model by changing the number of transformer layers in ViT. When the number of layers was set to four and eight, the average accuracies of the model output were 94.65% and 94.68%, respectively, for Dataset A. The most essential structure in a transformer is multi-head attention. MSA was set to four and eight heads, respectively, in this study, and the experimental results showed that the accuracy of the eight-head MSA was improved. Another vital structure in the transformer structure is the MLP. The neurons in each hidden layer receive information from all the neurons in the adjacent preceding hidden layers and then output the information to all the neurons in the adjacent hidden layers after processing. We set the hidden layers of the MLP to 128, 256, and 512 and experimentally demonstrated that increasing the number of hidden layers can improve the accuracy of the model output. We also investigated the effect of the output channels on model performance. When the output channels were changed from 128 to 256, the average training time increased from 4.32 s to 9.87 s, with no significant improvement in accuracy. The deficiency of TranSegNet is that it requires sufficient memory space to compute the multi-head self-attention feature matrix, and because of the limitation of GPU memory, we could not precisely prove the effect of its parameters on the model. Here, the network training batch size can only be set to a maximum of eight. When the batch size was changed from two to four to eight, the accuracy improved, but not significantly. Finally, considering the complexity, efficiency, and accuracy of the model, the final parameters of TranSegNet used were set as follows: out channels = 128, head num = 8, MLP dim = 128, block num = 4, patch dim = 16, and batch size = 8.

The loss function in this study consisted of a weighted cross-entropy and dice function, as shown in Equation (9). To verify the validity of these two metrics, we researched training TranSegNet using only cross-entropy loss and dice loss. The results indicate that the average accuracy of the training using cross-entropy and Dice coefficients was 0.9256 and 0.8751, respectively, which is significantly worse than the combined result of 0.9456 (Table 1). This is because cross-entropy loss only considers the loss in a microscopic sense and ignores whether the adjacent pixels are bounded. The stability of the dice loss cannot be guaranteed when the statistical distribution of labels is unbalanced. To determine the optimal weight parameters in the loss function Equation (9), a series of experiments were conducted. Consequently, we achieved the best overall performance by combining the cross-entropy and dice functions and setting the weight parameters α and β to 0.5.

Additionally, we designed the input and output of the ViT module to be of the same size to ensure that it could be easily embedded in our network. This design will facilitate further TranSegNet modifications in the future. Compared with other transformer-encoded models, when the transformer is applied to natural language processing or image processing tasks, it lacks translation invariance and local perception, which may be improved by pre-training on large-scale datasets. Given that the feature information of the laminar structure of retinal OCT images is relatively stable, the lightweight ViT in our study did not use extra position-encoded information, but instead performed accurate localization by preserving position indices of the pooling layer and the long jump connections to enhance spatial information in the network backbone. Thus, by removing the position-encoding component, the pre-training time and the model size are reduced while maintaining comparable model performance. We demonstrated the feasibility of our method and its strengths for retinal OCT image segmentation using various metrics of different experimental results. 

## 5. Conclusions

In this study, a hybrid neutral network architecture, TranSegNet, was proposed for OCT retinal image segmentation. The core of TranSegNet is the CNN-ViT encoder, which is based on an improved U-shaped network architecture to extract important features automatically and introduces a lightweight vision transformer with multi-head convolutional attention to model long-range dependencies. TranSegNet can be applied effectively to small-scale datasets without pre-training. Two retinal OCT datasets were used in this study to evaluate the performance for segmenting different retinal morphologies: a self-made dataset from a lab-built OCT system and a public SD-OCT dataset from DME patients. Compared with FCN, U-Net, SegNet, and TransUnet, TranSegNet demonstrated better performance and generalization ability in the segmentation of healthy retinas affected by blood vessels, diseased retinas with morphological changes, and fluid accumulation, exhibiting high consistency among metrics. TranSegNet has the potential for further improvement, and its end-to-end design makes it easy to use for OCT image segmentation. However, due to limited hardware resources, further research is needed to explore the performance of TranSegNet on larger datasets and to investigate methods to improve translation invariance. Overall, TranSegNet demonstrates promising results in OCT retinal image segmentation and the potential for further improvement to explore other possible extensions to improve the diagnosis of retinal diseases.

## Figures and Tables

**Table 1:** Experimental results of different segmentation methods.

	Acc (%)	HD (μm)	FLOPs (G)	Para (M)	Training Time (s)
FCN (2014)	92.53 ± 1.30	6.92 ± 0.98	194.71	31.90	219.30
U-Net (2015)	93.18 ± 0.65	7.53 ± 2.49	516.46	16.42	406.60
SegNet (2015)	93.45 ± 0.21	5.63 ± 1.12	255.14	19.41	280.82
TransUnet (2021)	93.36 ± 0.40	9.30 ± 1.10	383.96	42.66	352.03
TranSegNet	94.64 ± 0.06	5.29 ± 0.77	195.48	18.58	218.11

**Table 2:** Evaluation of different segmentation methods for Dataset A.

	%	BG	NFL	GCL+IPL	INL	OPL	ONL+IS	OS	OPR	RPE
FCN	Precision	99.73	80.04	97.51	75.87	69.13	**98.24**	75.88	87.19	75.61
	Recall	97.09	93.52	90.12	**93.15**	80.97	92.86	86.76	81.59	92.78
	DSC	98.42	86.26	94.13	83.63	74.58	95.48	80.96	84.30	83.32
Unet	Precision	**99.82**	81.89	98.11	84.61	72.43	98.30	78.40	86.26	79.52
	Recall	97.47	95.37	93.64	88.87	**85.44**	94.04	84.73	87.44	93.25
	DSC	98.63	88.12	95.83	86.69	78.82	96.13	81.44	86.85	85.84
SegNet	Precision	99.80	80.17	97.25	87.83	69.60	96.96	**78.49**	81.94	**81.14**
	Recall	97.48	95.93	93.89	85.35	83.67	93.26	81.76	87.04	91.91
	DSC	98.63	87.76	96.04	86.57	76.80	95.08	81.12	84.41	86.90
TransUnet	Precision	99.42	81.18	97.38	88.07	67.53	97.00	73.25	84.71	78.58
	Recall	97.08	95.75	93.34	82.69	85.09	93.76	88.66	**88.12**	89.98
	DSC	98.23	**88.45**	95.32	85.29	75.30	95.35	80.22	86.38	83.90
TranSegNet	Precision	99.75	**82.18**	**97.91**	**89.29**	**77.40**	98.21	77.98	**91.30**	80.46
	Recall	**97.99**	**96.36**	**94.33**	91.29	85.37	**94.56**	**89.83**	85.32	**95.56**
	DSC	**98.86**	87.76	**96.64**	**90.28**	**80.27**	**96.35**	**82.14**	**88.21**	**87.36**

**Table 3:** Evaluation of different segmentation methods for Dataset B.

	%	BG	NFL	GCL +IPL	INL	OPL	ONL +IS	ISE	OS-RPE	Choroid	Fluid
FCN	Precision	**99.74**	68.9	93.15	66.48	57.48	90.92	89.14	82.5	99.9	10.05
	Recall	96.6	92.29	83.9	**68.7**	78.9	83.83	88.17	88.6	99.03	5.37
	DSC	98.15	79.26	88.29	69.3	66.58	87.23	88.65	85.44	99.49	9.66
U-net	Precision	99.59	70.3	**93.19**	62.37	51.43	**91.70**	**91.71**	80.96	**99.96**	23.64
	Recall	96.77	91.4	83.27	67.22	78.79	81.85	89.12	91.21	99.04	14.23
	DSC	98.15	79.47	87.95	69.01	62.22	86.94	90.39	85.78	99.48	23.20
SegNet	Precision	99.73	79.14	89.27	56.27	62.6	89.28	86.79	78.14	99.86	19.81
	Recall	97.57	92.08	84.55	69.24	74.81	83.33	87.78	**92.31**	**99.29**	3.11
	DSC	98.69	85.12	86.85	62.08	68.16	87.58	87.28	85.95	99.52	5.38
TransUnet	Precision	93.97	76.99	91.66	67.95	67.06	87.13	89.28	86.57	99.81	39.73
	Recall	97.28	89.32	83.63	64.62	66.68	91.70	92.37	91.24	97.97	12.12
	DSC	95.6	82.7	87.46	66.24	66.87	89.36	90.36	86.84	98.88	19.95
TranSegNet	Precision	99.47	**81.53**	92.88	**68.73**	**67.69**	91.45	88.17	**86.57**	**99.96**	**47.56**
	Recall	**98.24**	**93.05**	**86.99**	**70.35**	**79.10**	**92.78**	**93.55**	92.20	99.26	**30.17**
	DSC	**98.86**	**86.47**	**89.84**	**72.20**	**68.33**	**89.84**	**91.36**	**88.84**	**99.57**	**29.49**

## Data Availability

The DUKE dataset is available online: https://people.duke.edu/~sf59/ (accessed on 10 September 2022). Our self-made dataset will be publicly available at https://www.kaggle.com/datasets/ (accessed on 4 March 2023).
